# 43 Unemployed Burn Survivors Experience Significantly Worse Long-term Psychosocial and Functional Outcomes

**DOI:** 10.1093/jbcr/iraf019.043

**Published:** 2025-04-01

**Authors:** Deborah Choe, Ayumi Saito, Andrew Humbert, Kimberly Roaten, Karin Blen, Jeffrey Schneider, Juan Herrera-Escobar, Haig Yenikomshian

**Affiliations:** University of California Keck School of Medicine; University of Washington; University of Washington; University of Texas Southwestern; Los Angeles General Medical Center; Spaulding Rehabilitation Hospital, Harvard Medical School; Brigham and Women’s Hospital, Harvard Medical School; University of California Keck School of Medicine

## Abstract

**Introduction:**

Long-term recovery among burn survivors is multifactorial, including characteristics such as total body surface area burned and mechanism of injury. Various preinjury factors such as a history of depression and anxiety are also associated with impaired long-term recovery. One social factor that has not been well studied is pre-injury employment status on patient-reported outcomes post-injury. Understanding the longitudinal health impact of employment status among burn survivors is critical to improving public health outcomes and long-term recovery. In this study, we aimed to compare psychosocial and functional domains between adults who were employed and unemployed before burn injury to examine the association with long-term outcomes.

**Methods:**

Adult burn survivors participating in a national longitudinal, multicenter patient-reported outcome database between 2015-2023 were included. Participants were divided into two groups: those who were employed at the time of injury and those who were unemployed. Survey responses using the PROMIS scales (Pain, Anxiety, Depression, Fatigue, Ability to Participate in Social Roles, and Physical Function); Community Integration Questionnaire (Social only); PTSD (PCL-5); and Satisfaction with Life (SWL) scale at 6-, 12-, and 24-months post-burn injury were analyzed. Associations between pre-burn employment status and long-term outcome scores were analyzed using mixed-effects models while adjusting for sex, age, TBSA burn size, presence of the COVID-19 pandemic, and time after injury.

**Results:**

A total of 814 participants were included, of which 635 were employed and 179 were unemployed pre-burn injury. Compared to the employed group, the unemployed group reported significantly worse Anxiety T-scores (2.97 points higher, p< 0.001), Depression T-scores (3.02 points higher, p< 0.001), Fatigue T-scores (3.04 points higher, p=0.001), Ability to Participate in Social Roles T-scores (4.3 points lower, p< 0.001), Physical Function T-scores (5.03 points lower, p< 0.001), Community Integration total scores (1.15 points lower, p< 0.001), PTSD total scores (3.21 points higher, p< 0.01), and SWL total scores (2.96 points lower, p< 0.001) after adjusting for sex, age, burn size, presence of the COVID-19 pandemic, and time after injury.

**Conclusions:**

These findings indicate that adult burn survivors unemployed pre-burn injury experience significantly worse long-term psychosocial and functional outcomes post-burn injury compared to those employed pre-burn injury. Employment status may be an important social factor when identifying adult burn survivors who may benefit from greater psychological, vocational, and social resources to optimize long-term recovery and improve quality of life.

**Applicability of Research to Practice:**

Resources and follow-up services focused on enhancing psychosocial and physical recovery should be targeted to this at-risk group of adults living with burn injuries.

**Funding for the Study:**

The contents of this abstract were developed under a grant from the National Institute on Disability, Independent Living, and Rehabilitation Research. NIDILRR is a Center within the Administration for Community Living (ACL), Department of Health and Human Services (HHS). The contents of this abstract do not necessarily represent the policy of NIDILRR, ACL, or HHS, and you should not assume endorsement by the Federal Government.

